# A Novel Prognostic Signature Associated with Immunotherapeutic Response for Hepatocellular Carcinoma

**DOI:** 10.3389/fsurg.2022.905897

**Published:** 2022-07-05

**Authors:** Xinmin Jin, Jinhuan Wang

**Affiliations:** ^1^ Department of Clinical Medical, Qingdao University Medical College, Qingdao China; ^2^ Department of Pharmacy, The Affiliated Hospital of Qingdao University, Qingdao China

**Keywords:** hepatocellular carcinoma, necroptosis, prognostic signature, immune infiltration, immunotherapy

## Abstract

**Background:**

Although accumulating literature has validated that necroptosis plays a prominent role in the tumorigenesis and progression of various malignant cancer, its mechanism in hepatocellular carcinoma (HCC) is poorly understood. Therefore, in the present study, we want to study the impact of necroptosis-related genes on the prognosis and microenvironment-infiltrating immunocytes and the effect of immunotherapy on patients with HCC.

**Methods:**

The necroptosis-related genes were obtained by reviewing the available published literature; we then evaluated the effects of the prognostic genes on the relative abundance of microenvironment infiltrated immunocytes. After construction of the Risk Score Signature, we evaluated the prognostic value and the effects on immune cells infiltrating the tumor microenvironment (TME). Combining the available data on immunotherapy, we also investigated the impact on anti-PD-L1-based immunotherapy.

**Results:**

A comprehensive study of the published literature confirmed that 22 genes are related to necroptosis. Among them, 10 genes were related to the prognosis of the HCC cohort in The Cancer Genome Atlas (TCGA) and had a multifaceted influence on TME. We obtained the Risk Score Signature by Lasso regression. Furthermore, we also corroborated the correlation between the Risk Score Signature and tumor-infiltrating immune cells in the TME. Next, in the study of the correlation between the Signature and immunotherapy, we found that the Signature was significantly correlated with the reactivity of anti-PD-L1 immunotherapy. We also confirmed that the Risk Score Signature is a reliable and efficient independent prognostic marker of HCC.

**Conclusion:**

We established a novel and effective prognostic model for patients with HCC, which is markedly related to the TME and immune infiltration in HCC and can also predict immunotherapeutic response and prognosis.

## Introduction

Programmed cell death, also called apoptosis, is a critical physiological process in the homeostasis of pluricellular organisms. However, if this process is out of control, aberrant cell death may lead to tissue injury, immune disorders, various diseases, and even tumors. As generally known, apoptosis is a type of caspase-dependent programmed cell death; necroptosis has received considerable attention in recent years as a caspase-independent programmed form of cell death.

**Figure 1:**
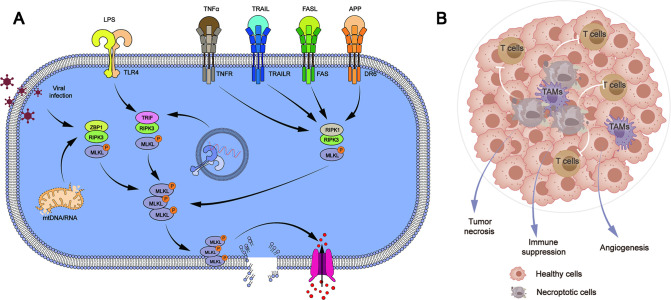
Necroptosis pathway (**A**) and its role in cancer immunity (**B**)

Although both are potential mechanisms for cell death, necroptosis is considered different from apoptosis. The pathogenic process of human malignant tumors is influenced by the defect or mutation of necroptosis-related genes. The defects of necroptosis-related genes have been discovered in the cells of chronic lymphocytic leukemia patients. Patients with non-Hodgkin lymphoma have mutations in necroptosis-related genes and aberrant activation of necroptosis-related pathways (1). Mutations in critical necroptosis genes have been identified to speed up tumor cell proliferation and migration in epidermal malignancies (2). Necroptosis has been shown to hasten cancer cell death or boost tumor cell susceptibility to anticancer drugs (3–6). These findings show that the mechanism of necroptosis might be a potential target for tumor therapy (Figure 1).

Based on data from GLOBOCAN 2018, according to ICD-10, there are approximately 792,031 new cases of HCC in the world with the age-standardized incidence rate (ASR) of 8.9 per 100,000 person-year, ranking 6th in the incidence among all malignant tumors; the ASR for males was 13.4 per 100,000, ranking 5th in the incidence spectrum of malignant tumors in male patients, while the ASR for females was 4.7 per 100,000, ranking 9th in the incidence of malignant tumors in female patients (7). HCC is the seconnd leading cause of malignant tumor deaths and disability-adjusted life years (DALYs) worldwide (7). In 2017, HCC was the fifth largest cause of years of life lost (YLLs) and the seventh leading cause of disability-adjusted life years (DALYs) among all chronic diseases in China (8). Although the diagnosis and treatment technology of HCC has advanced significantly in recent years, the age-standardized 5-year net survival increased from 11.7% in 2000–2004 to 14.1% in 2010–2014 (9). However, the 5-year survival rate of patients with early-stage HCC [Barcelona Clinic Liver Cancer (BCLC) stage 0/A] who underwent radical treatment may be as high as 69.0%–86.2% (10). Therefore, the early detection of HCC is extremely critical.

Using bioinformatics tools, we hope to investigate the expression and prognostic value of necroptosis-related genes in HCC to develop an independent prediction model for HCC, allowing us to detect and treat HCC at the earliest possible stage.

## Materials and Methods

### Data Acquisition

UCSC Xena (https://xenabrowser.net/datapages/) was used to retrieve TCGA RNA-seq datasets, copy number variation (CNV) data, and clinical data of liver hepatocellular carcinoma (LIHC); there were 374 HCC tumors and 50 normal tissues. The independent cohorts (LIRI-JP) in the International Cancer Genome Consortium (ICGC) (https://dcc.icgc.org/) database were employed in our research, including 240 cases of HCC tissues and 197 cases of normal tissues. Primary processing of data was performed by the R package tinyarray.

### Collection and Analysis of Necroptosis-Related Genes

A thorough search for all relevant literature yielded necroptosis-related genes (11–21) (RIPK3, RIPK1, MLKL, NDRG2, CXCL1, BIRC2, TLR3, TLR2, ALDH2, TNFRSF1A, TLR4, CYLD, PGAM5, NR2C2, ZBP1, HMGB1, BIRC3, USP22, FADD, TRAF2, EZH2, and TNF). The Mann–Whitney *U* test revealed that 18 genes were differently expressed in HCC samples compared to normal samples. Survival analysis of the above 18 genes was evaluated by univariate Cox regression analysis using the survival R package (22); CNV was analyzed using the maftools R package. Enrichment analysis and correlation analysis between genes were performed; we also explored the relationship between prognosis-related genes and the tumor microenvironment, immune cell infiltration, and immune checkpoint genes (23).

### Constructing and Validating the Risk Score Signature

A multigene-based Risk Score Signature for predicting prognosis was constructed using Lasso regression to further identify prognosis-related genes in HCC patients. The sum of the products of each gene's expression value and the accompanying coefficients was used to calculate each patient's risk score. Patients will be separated into low-risk and high-risk groups according to the cutoff calculated by the R package survminer. The survival packages were used to test the prediction efficacy of the Risk Score Signature on prognosis. To assess the Risk Score Signature's prediction ability, timeROC packages were used to plot 1-, 3-, and 5-year ROC curves. We utilized the tinyarray packages to investigate the relationship between the Risk Score Signature and necroptosis-related genes.

### Risk Score Signature Combined With Immune System

We evaluate the relationship between the Risk Score Signature and immune cell infiltration based on an article containing 28 categories of immune cells and their marker genes (23, 24). As immune checkpoints become more important for tumor progression, treatment, and prognosis, we investigated the specific correlation of Risk Score Signature with major immune checkpoints. The distribution of key genes at the single-cell level in HCC was then validated by TISCH databases (http://tisch.comp-genomics.org/) (25, 26). The IMvigor210 cohort was utilized to investigate the predictive efficacy of Risk Score Signature on anti-PD-L1 immunotherapy (27). The IMvigor210 cohort, which is made up of urothelial carcinoma patients who were given anti-PDL1 antibody atezolizumab, was widely utilized to predict patient response to immunotherapy.

### Statistical Analysis

R software was mostly used to analyze and illustrate the data (R version 4.1.2). To construct ROC curves, Kaplan–Meier curves, and forest plots for Cox regression, the timeROC, survminer, and survival R packages were used. The mutation landscape was examined by the maftools R package. Regplot and survival were employed for the construction and presentation of the Nomogram. The Wilcoxon rank-sum test was used to compare the expression levels of the two groups. Statistical significance was defined as *P* < 0.05.

## Results

### Expression and Mutation of Necroptosis-Related Genes in HCC

**Figure 2:**
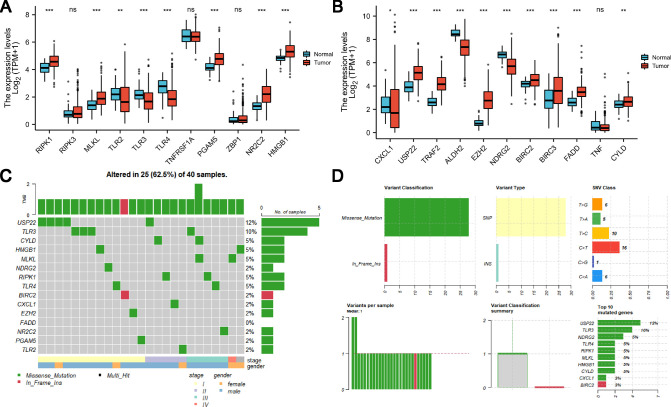
Expression and genetic mutation of necroptosis-related genes in HCC. (**A** and **B**) Expression of necroptosis-related genes in HCC. (**C** and **D**) Incidence of necroptosis-related gene mutations and their categorization in HCC.

The relative expression levels of necroptosis-related genes in tumor tissue and normal tissue were examined as shown in Figures 2A,B; we found that 18 of these genes showed changes in gene expression at the mRNA level. RIPK1, MLKL, PGAM5, NR2C2, HMGB1, USP22, TRAF2, EZH2, BIRC2, BIRC3, FADD, and CYLD are necroptosis-related genes with elevated expression levels in HCC. In normal liver tissues, TLR2, TLR3, TLR5, CXCL1, and NDRG2 were all upregulated (Figures 2A,B). In the TCGA HCC cohort, mutations in necroptosis-related genes occurred in 40 patients with HCC, there was a preponderance of stage I patients, and male patients were in the majority. USP22 recorded the highest frequency of mutations (Figure 2C). Mistranslation mutation was the most common variant categorization, whereas the most prevalent variation type was single nucleotide polymorphism **(**SNP**)**. When compared to the other SNV classes, C > T had the highest frequency (Figure 2D).

### Identification, Functional Enrichment, and Effect on TME-Infiltrating Cells of Prognosis-Related Genes

**Figure 3:**
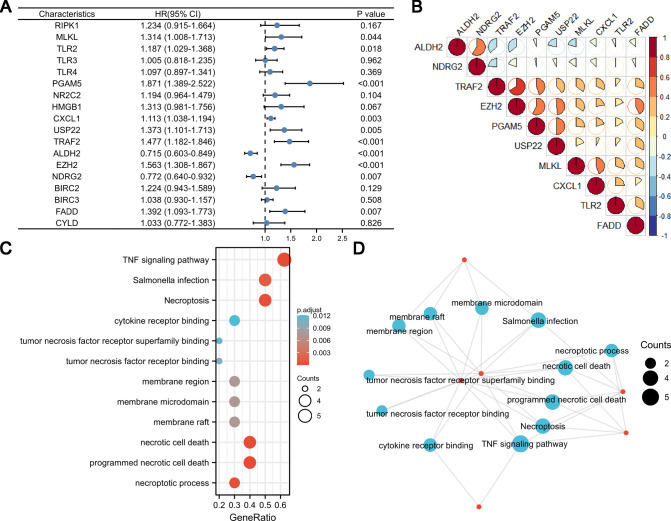
Univariate Cox analysis, correlation analysis, and function enrichment analysis of necroptosis-related genes. (**A**) Forest map showing 18 necroptosis-related genes for HCC identified by univariate Cox analysis. (**B**) Correlation visualization matrix displaying the pairwise correlation between the 10 necroptosis-related genes. (**C** and **D**) Function enrichment analysis of necroptosis-related genes.

**Figure 4:**
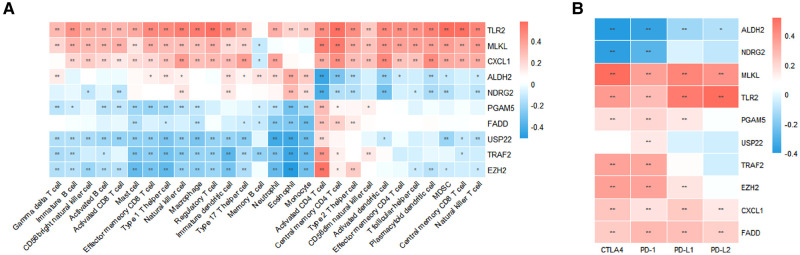
Characterization of TME immune cell infiltration. (**A**) Correlation between prognostic necroptosis-related genes and 28 TME-infiltrating cell types. (**B**) Relationship between prognostic necroptosis genes and immune checkpoint molecules.

Using univariate Cox analysis, we found that 10 of the 18 necroptosis-elated genes differentially expressed in HCC were related to the prognosis of HCC (Figure 3A). Various degrees of correlation were revealed between each other; PGAM5 had the strongest correlation with EZH2, whereas NDRG2 and TLR2 had negligible correlation (Figure 3B). Enrichment analysis was used to characterize the function of these prognostic genes. Under the criteria of *P*.adj < 0.05 and *q*-value < 0.2, a total of 3 MF terms, 158 BP terms, 3 CC terms, and 23 KEGG pathways were significantly enriched (Figure 3C). According to the common genes in the enriched pathway, we networked the results of functional enrichment (Figure 3D). We explored the influence of prognostic genes on immune cells identified in the TME, as well as immunological checkpoints, to further understand the connection between prognostic genes and tumor immunity. TLR2, MLKL, and CXCL1 were discovered to be highly connected with the majority of infiltrating immune cells in the TME, and these three genes were also strongly linked to immunological checkpoints (CTLA4, PD-1, PD-L1, and PD-L2). Except for ALDH2 and NDRG2, activated CD4+ T cells were positively linked with almost all prognostic genes (Figures 4A,B).

### Constructing and Validating the Risk Score Signature

**Figure 5:**
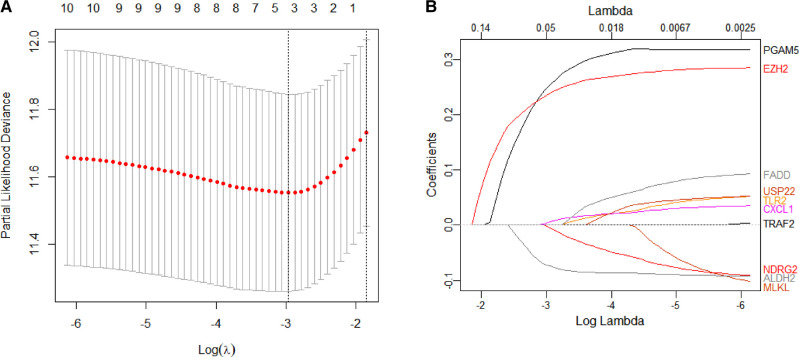
(**A** and **B**) Coefficient and partial likelihood deviance of the Risk Score Signature.

**Figure 6:**
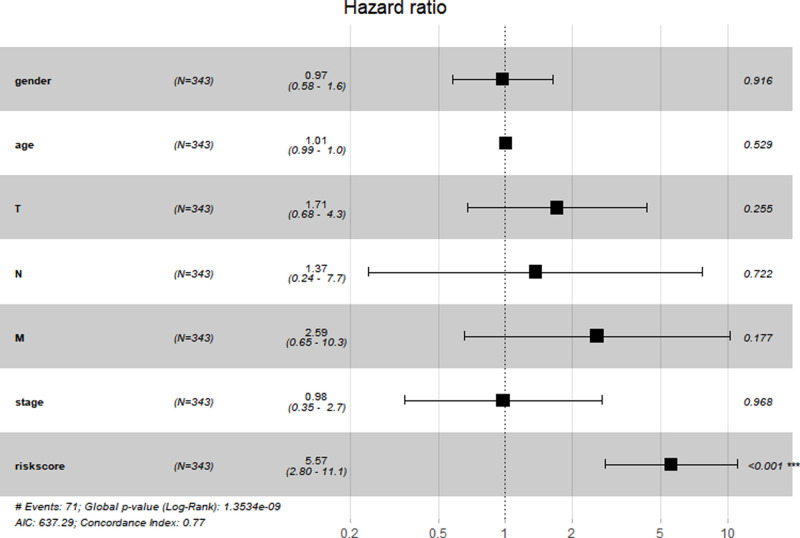
Prognostic value of the Risk Score Signature. Forest plot showing that the Risk Score Signature was an independent prognostic biomarker.

**Figure 7:**
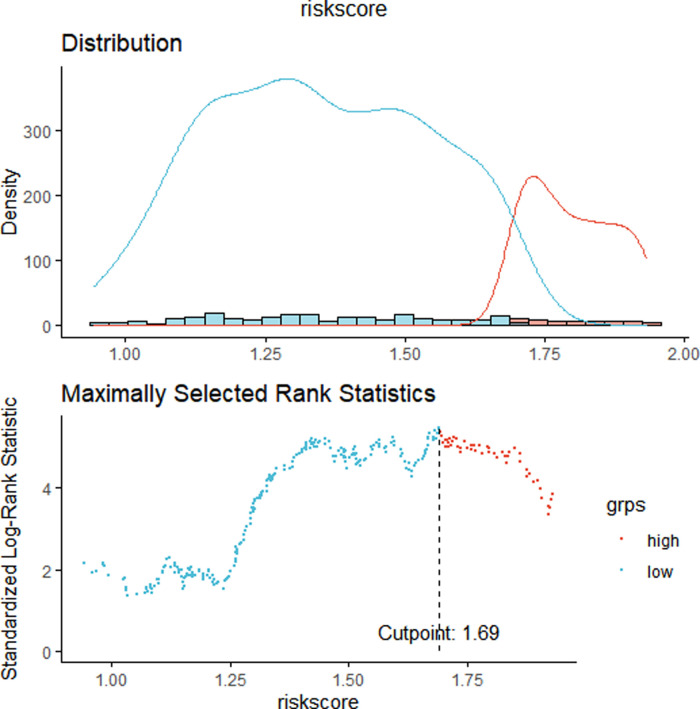
Determination of cutoff.

**Figure 8:**
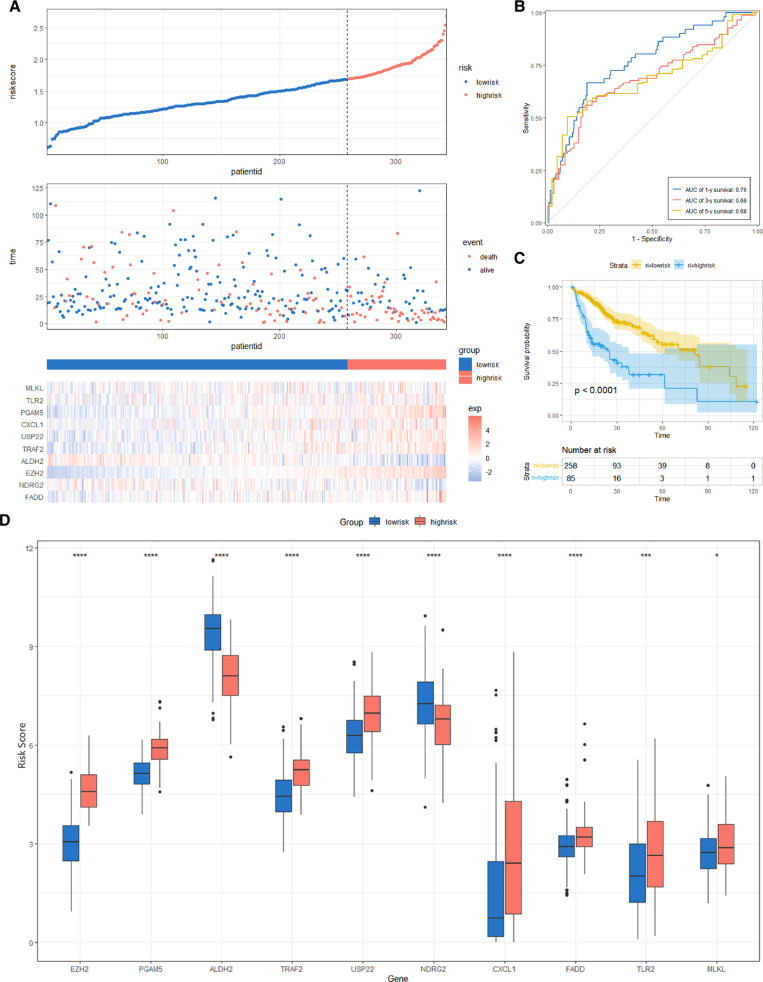
Risk score analysis, prognostic performance, and survival analysis of the Risk Score Signature in the TCGA cohort. (**A**) Risk score and survival time distribution of patients and gene expression of necroptosis-related genes in the Risk Score Signature. (**B** and **C**) Overall survival curve and ROC curve of the Signature. (**D**) Key molecules expressed in the low- and high-risk groups.

**Figure 9:**
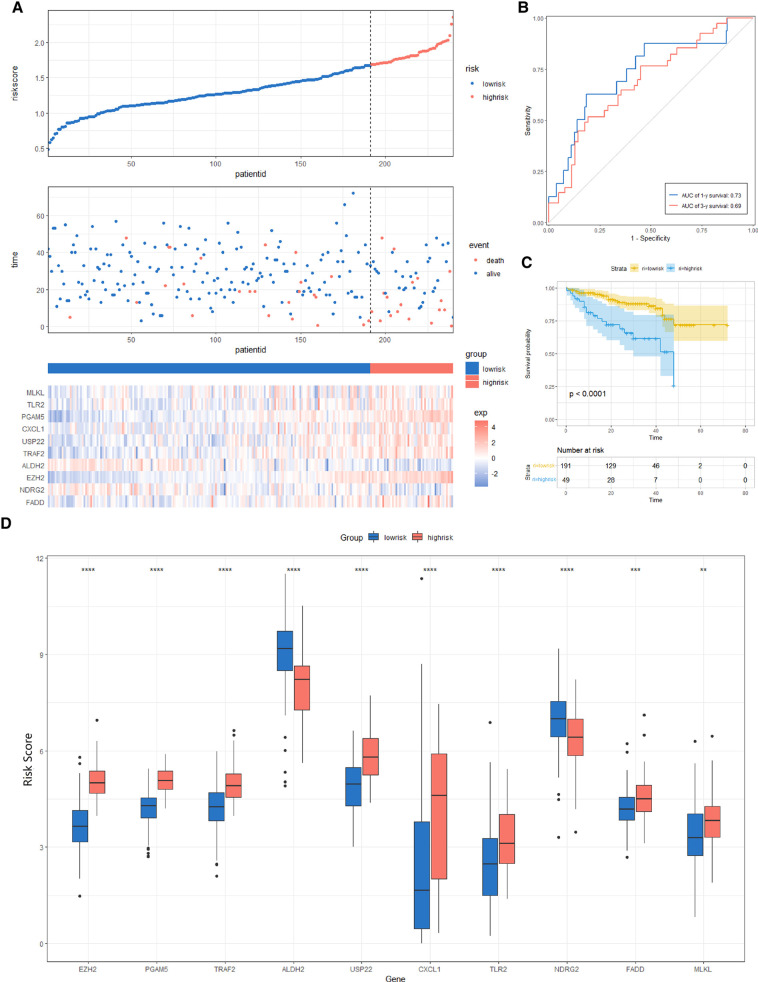
Risk score analysis, prognostic performance, and survival analysis of the Risk Score Signature in the ICGC cohort. (**A**) Risk score and survival time distribution of patients as well as gene expression of necroptosis-related genes in Risk Score Signature. (**B** and **C**) Signature's overall survival and ROC curves. (**D**) Expression of key molecules in the low- and high-risk groups.

**Figure 10:**
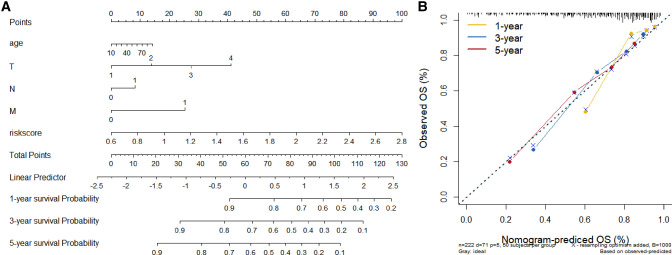
Establishment and evaluation of Nomograms. (**A**) 1-, 3-, and 5-year Nomograms for predicting OS of HCC. (**B**) Nomogram's calibration curves for predicting 1-, 3-, and 5-year OS in the TCGA cohort.

We constructed a Risk Score Signature by Lasso regression analysis of 10 necroptosis-related genes related to the prognosis of HCC based on univariate Cox analysis. The risk score equations are formulated as follows: risk score = (0.243) *PGAM5+ (0.0024) *CXCL1+ (-0.0708) *ALDH2+ (0.2325) *EZH2. Partial likelihood deviance for the LASSO coefficient profiles of the 10 prognostic genes is demonstrated in Figures 5A,B. As shown in Figure 6, univariate Cox analysis shows that risk score can serve as an independent diagnostic biomarker. According to the cutoff calculated by the R package survminer, patients were separated into high-risk and low-risk groups (Figure 7). Almost all necroptosis-related genes associated with prognosis were substantially expressed in the high-risk group, except NDRG2 and ALDH2 (Figures 8A,D). In the 1-year, 3-year, and 5-year ROC curves, the values of the area under the ROC curve were 0.75, 0.68, and 0.68, respectively (Figure 8B). The Kaplan–Meier survival curve revealed that individuals in the low-risk group lived substantially longer than those in the high-risk group, indicating that the Risk Score Signature had a strong prognostic value (Figure 8C). We employed the ICGC cohort (Project LIRI-JP) as an external validation set to validate the Risk Score Signature further. According to the same cutoff value as the TCGA cohort, patients from the ICGC cohort were separated into high-risk and low-risk groups (Figure 9A). In 1-year and 3-year ROC curves for the ICGC cohort, the values of the area under the ROC curve were 0.73 and 0.69, respectively (Figure 9B). To increase the accuracy of Risk Score Signature prediction, we created a Nomogram, which we integrated with other characteristics to assess the probability of HCC patients surviving (Figure 9A). The calibration diagram for 1-, 3-, and 5-year OS is shown in Figure 10B.

### Role of Risk Score Signature in the TME Cell Infiltration and Immunotherapeutic Response

**Figure 11:**
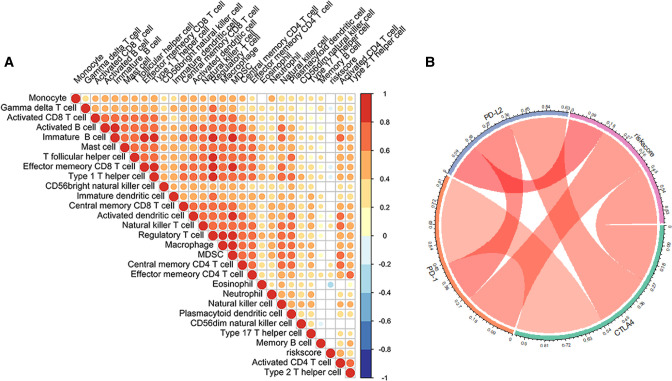
Role of the Risk Score Signature in the TME cell infiltration and immune checkpoint molecule. (**A**) Relationship between the Risk Score Signature and the infiltration of 28 TME cells. (**B**) Correlation between the Risk Score Signature and immune checkpoint molecule.

**Figure 12:**
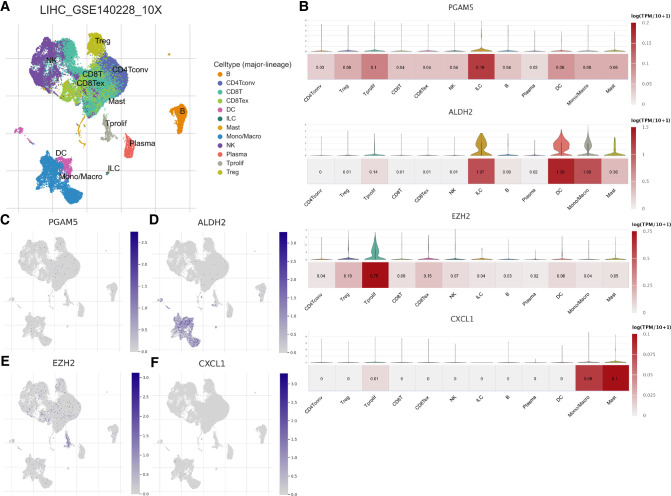
Distribution of key genes constituting the Risk Score Signature at the single-cell level. (**A**) Immune cells clustered in GSE140228. (**B**) Violin map and heat map of the expression of key genes in clustered immune cells. Umap map of the expression of PGAM5 (**C**), ADH2 (**D**), EZH2 (**E**), and CXCL1 (**F**) in clustered immune cells.

**Figure 13:**
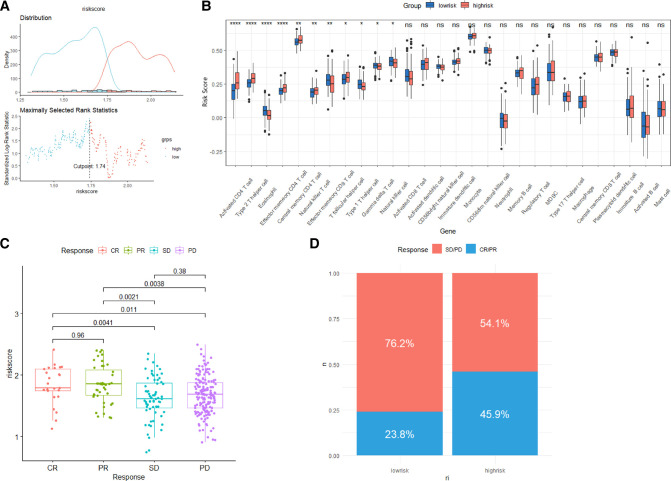
Role of the Risk Score Signature in immunotherapeutic responses. (**A**) Determination of cutoff. (**B**) Differences in 28 TME infiltration cells between low- and high-risk groups. (**C**) Relationship between the Risk Score Signature and anti-PD-L1 clinical response. (**D**) Proportion of patients responding to anti-PD-L1 therapy in low- or high-risk groups.

We assessed the association between Risk Score Signature and 28 infiltrating immune cells to evaluate the connection between Risk Score Signature and TME and discovered that the Risk Score Signature was significantly negatively connected to eosinophils but considerably positively related to effector memory CD4 T cells, central memory CD4 T cells, and natural killer T cells (Figure 11A). We also discovered that the expression of immune checkpoint protein was strongly connected with the Risk Score Signature (Figure 11B). To better understand the relationship between Risk Score Signature and immune cells in liver cancer, we analyzed the expression of key genes at the single-cell level in HCC; 12 types of immune cells were clustered. Finally, we found that among the four key genes, PGAM5 and CXCL1 were less expressed in immune cells, while ALDH2 was closely related to DC, ILC, and mono/macro and EZH2 was mainly expressed in Tprolif (Figure 12). We also evaluated the clinical relevance of Risk Score Signature and the effectiveness of anti–PD-L1 immunotherapy and discovered that following immunotherapy, division of the patients into two groups according to the cutoff calculated by the survminer package (Figure 13A), activated CD4 T cells were the immune cells most closely connected with risk score in patients receiving immunotherapy (Figure 13B), and patients in the high-risk group seemed to be more responsive (Figures 13C,D).

## Discussion

Hepatocellular carcinoma is one of the most common malignancies around the world, with the second-highest fatality rate among all cancers in China (28). Chronic hepatitis virus infection, alcoholism, autoimmune liver disease, nonalcoholic steatohepatitis, drug-induced liver injury, and aflatoxin are all risk factors for HCC (29). The clinical treatment of HCC includes liver transplantation, surgical resection, chemotherapy, radiotherapy, interventional therapy, molecular targeted therapy, and immunotherapy. Although surgical resection is currently the most effective treatment for HCC, the resectable rate of HCC at the time of diagnosis is only 20%–30%. Because of intrahepatic and extrahepatic metastases, most HCC patients are unable to undergo radical surgery. In previous research, the immune microenvironment of the liver has been proven to be intimately linked to the tumorigenesis and development of HCC. Various tumor-related immune cells such as tumor-infiltrating lymphocytes (TILs), CD8 + cytotoxic T lymphocytes (CTLs), tumor-associated macrophages (TAMs), regulatory T (Treg) cells, and marrow-derived suppressor cells (MDSCs) play a role in HCC progression (30, 31). We conducted this study to predict better the prognosis of patients with HCC and their response to immunotherapy.

By analyzing and summarizing existing studies, we found 22 genes associated with necroptosis, of which 18 genes were differently expressed between HCC and normal tissues. To be able to further clarify the impact of these genes on the prognosis of patients with HCC, we used univariate Cox analysis to obtain 10 prognostic necroptosis-related genes. We performed Lasso regression analysis to find genes that may be used as potential markers, and we came up with a Risk Score Signature that included PGAM5, CXCL1, ALDH2, and EZH2. We used an external dataset from the ICGC database to establish the validity and reliability of the Risk Score Signature. The findings indicate that the Signature is both reliable and effective. Whether in the TCGA cohort or the ICGC cohort, the Signature can be used as an effective independent predictor to predict the prognosis of HCC. The high-risk group, divided according to the risk score, is significantly correlated with adverse outcomes. Although not listed in the Nomogram, some researchers found that MVI, satellite status, margin status, and other factors also have an important impact on the prognosis of patients with HCC (32).

We also concluded that the Risk Score Signature was presented in relation to immune cell infiltration in the TME as well as levels of immune checkpoint proteins. Although the trends differ, the analysis of the relationship with immunotherapy reveals that the Risk Score Signature has a significant correlation in predicting responsiveness to immunotherapy. Case reports have shown that in individual patients, using VEGFR TKI alone or combined chemotherapy can significantly shrink the tumor before surgery or other deterministic local treatment. Combined with our findings, we believe that preoperative immunotherapy may improve the survival of patients preparing for liver transplantation, especially those with high-risk scores.

## Conclusion

In conclusion, the Risk Score Signature we established based on necroptosis-related genes may accurately and reliably predict the prognosis of HCC patients. We discovered that necroptosis-related genes play an essential role in the incidence, progression, and immune tolerance of HCC by analyzing the connection between the Risk Score Signature and immune cell infiltration, immune checkpoint, and immunotherapeutic response, which means that our finding could become a potential therapeutic target for HCC.

## Data Availability

The original contributions presented in the study are included in the article/Supplementary Material; further inquiries can be directed to the corresponding authors.
